# Inhibition of fucosylation in human invasive ductal carcinoma reduces E‐selectin ligand expression, cell proliferation, and ERK1/2 and p38 MAPK activation

**DOI:** 10.1002/1878-0261.12163

**Published:** 2018-03-30

**Authors:** Mylène A. Carrascal, Mariana Silva, José S. Ramalho, Cláudia Pen, Manuela Martins, Carlota Pascoal, Constança Amaral, Isabel Serrano, Maria José Oliveira, Robert Sackstein, Paula A. Videira

**Affiliations:** ^1^ UCIBIO Departamento Ciências da Vida Faculdade de Ciências e Tecnologia Universidade Nova de Lisboa Portugal; ^2^ CEDOC Chronic Diseases Research Center NOVA Medical School/Faculdade de Ciências Médicas Universidade Nova de Lisboa Portugal; ^3^ Departments of Dermatology and Medicine Brigham & Women's Hospital Boston MA USA; ^4^ Harvard Medical School Program of Excellence in Glycosciences Boston MA USA; ^5^ Centro Hospitalar de Lisboa Central EPE – Serviço de Anatomia Patológica Lisbon Portugal; ^6^ Hospital de Cascais Portugal; ^7^ New Therapies Group INEB‐Institute for Biomedical Engineering Porto Portugal; ^8^ Instituto de Investigação e Inovação em Saúde Universidade do Porto Portugal; ^9^ CDG & Allies – PPAIN Congenital Disorders of Glycosylation Professionals and Patient Associations International Network Caparica Portugal

**Keywords:** breast cancer, cell migration, fucosylation, proliferation, sialyl‐Lewis X/A

## Abstract

Breast cancer tissue overexpresses fucosylated glycans, such as sialyl‐Lewis X/A (sLe^X^ ^/A^), and α‐1,3/4‐fucosyltransferases (FUTs) in relation to increased disease progression and metastasis. These glycans in tumor circulating cells mediate binding to vascular E‐selectin, initiating tumor extravasation. However, their role in breast carcinogenesis is still unknown. Here, we aimed to define the contribution of the fucosylated structures, including sLe^X^ ^/A^, to cell adhesion, cell signaling, and cell proliferation in invasive ductal carcinomas (IDC), the most frequent type of breast cancer. We first analyzed expression of E‐selectin ligands in IDC tissue and established primary cell cultures from the tissue. We observed strong reactivity with E‐selectin and anti‐sLe^X^ ^/A^ antibodies in both IDC tissue and cell lines, and expression of α‐1,3/4 FUTs FUT4, FUT5, FUT6, FUT10, and FUT11. To further assess the role of fucosylation in IDC biology, we immortalized a primary IDC cell line with human telomerase reverse transcriptase to create the ‘CF1_T cell line’. Treatment with 2‐fluorofucose (2‐FF), a fucosylation inhibitor, completely abrogated its sLe^X^ ^/A^ expression and dramatically reduced adherence of CF1_T cells to E‐selectin under hemodynamic flow conditions. In addition, 2‐FF‐treated CF1_T cells showed a reduced migratory ability, as well as decreased cell proliferation rate. Notably, 2‐FF treatment lowered the growth factor expression of CF1_T cells, prominently for FGF2, vascular endothelial growth factor, and transforming growth factor beta, and negatively affected activation of signal‐regulating protein kinases 1 and 2 and p38 mitogen‐activated protein kinase signaling pathways. These data indicate that fucosylation licenses several malignant features of IDC, such as cell adhesion, migration, proliferation, and growth factor expression, contributing to tumor progression.

**Abbreviations**
2‐FF: 2‐fluorofucose
BSA: bovine serum albumin
CFSE: 5,6‐carboxyfluorescein diacetate succinimidyl ester
E‐Ig: E‐selectin‐Ig chimera
ERK1/2: signal‐regulating protein kinases 1 and 2
FGF2: basic fibroblast growth factor
FUTs: fucosyltransferases
HBSS: Hank's balanced salt solution
HER2: human epidermal growth factor receptor 2
hTERT: human telomerase reverse transcriptase
IDC: invasive ductal carcinomas
IL: interleukin
MAPK: mitogen‐activated protein kinase
sLe^X/A^: sialyl‐Lewis X/A
TGF‐β1: transforming growth factor beta1
VEGFA: vascular endothelial growth factor

## 1 Introduction

Breast cancer is the major cause of cancer in women, accounting for ~ 30% of all newly diagnosed cancers worldwide. Invasive ductal carcinoma (IDC) is the most common type, making up nearly 70–80% of all breast cancer diagnoses. The 5‐year survival rate for breast cancer patients is almost 99% if the disease is detected in early stages. However, if the tumor has metastasized, the survival rate drastically decreases to 25% (Torre *et al*., 2015). These survival statistics prompt the need for new therapies that can prevent metastasis and the importance of better understanding its underlying mechanisms.

Metastasis to distant organs is a multistep process requiring growth of malignant cells, detachment from the primary tumor, penetration of the underlying basement membrane and surrounding tissue, intravasation and subsequent circulation through the blood and/or lymph vessels, and then emigration from the vasculature to colonize distant organs (Jacobs and Sackstein, 2011). Each step of this process requires different types of cell–cell interactions between cancer cells and the host microenvironment. Glycosylation is a post‐translational modification that is typically altered during malignant transformation and tumor progression (Carrascal *et al*., 2014; Ferreira *et al*., 2013; Pinho and Reis, 2015). One of the most important glycan terminal modifications is fucosylation, that is, the addition of fucose residues, catalyzed by fucosyltransferases (FUTs) that transfer fucose from the donor substrate, GDP‐fucose, to specific acceptors. The FUT3, 4, 5, 6, 7, and 9 transfer fucose in an α1,3‐ or α1,4‐linkage to GlcNAc in Gal‐GlcNAc‐ sequences. FUT1 and FUT2 catalyze fucose transfer in an α2‐linkage to terminal Gal residues in N‐ or O‐glycans, while FUT8 catalyzes α1,6‐linkages to the innermost asparagine‐linked (core) GlcNAc in N‐glycans (Miyoshi *et al*., 2012; Moriwaki and Miyoshi, 2010). Fucosylation yields the major vascular selectin ligands, the α1,3 or α1,4‐fucosylated glycan determinants, sialyl‐Lewis^X^ (sLe^X^) and sialyl‐Lewis A (sLe^A^). sLe^X^ and sLe^A^ are the principal binding determinants for vascular E‐selectin (Silva *et al*., 2011, 2012; Yago *et al*., 2010), which is typically induced on inflamed endothelium and constitutively expressed in bone microvessels (Schweitzer *et al*., 1996). E‐selectin engages its ligands expressed on blood circulating cells, capturing and decelerating the cells under flow and activating other cell mechanisms that promote tissue homing (Barthel *et al*., 2007; Tremblay *et al*., 2006; Yago *et al*., 2010). The relevance of E‐selectin‐mediated interactions in cancer metastasis has been reported in *in vivo* studies, wherein metastasis in mice was reduced when E‐selectin and/or E‐selectin ligand activity was blocked (Fukuda *et al*., 2000; Mannori *et al*., 1997). Furthermore, a positive correlation between the expression of sLe^X^ and sLe^A^ in primary breast cancer cells and tumor cell metastasis and/or invasion has been reported (Jeschke *et al*., 2005; Renkonen *et al*., 1997; Wei *et al*., 2010), suggesting that these glycan moieties also play an important role in early stages of the malignant transformation. However, the mechanistic basis of sLe^X/A^‐dependent pathogenesis of breast IDC is still unclear.

Biosynthesis of sLe^X^ and sLe^A^ requires the transfer of L‐fucose from GDP‐fucose to *N*‐acetylglucosamine located in a terminal type 1 or type 2 lactosamine, in an α‐1,4‐ or α‐1,3‐linkage, respectively, by the action of α1,4‐ or α1,3‐FUTs (Holmes *et al*., 1986; Oriol *et al*., 1999). α1,3/4 FUT expression has been reported to be involved in sLe^X^ and sLe^A^ synthesis in breast cancer (Ding and Zheng, 2004; Julien *et al*., 2011; Matsuura *et al*., 1998), as well as being involved in other cancer features as the epithelial–mesenchymal transition (Yang *et al*., 2013). Recently, fluorinated fucose (2‐FF) was shown to inhibit FUT activity, thus reducing cell surface fucosylated glycans such as Lewis antigens in colon carcinoma cells. This compound diminishes the adhesion of colon carcinoma cells to E‐selectin. In the same study, *in vivo* experiments showed that oral treatment of tumor‐bearing mice with 2‐FF resulted in inhibition of tumor outgrowth and xenograft tumor cell surface fucosylation (Okeley *et al*., 2013).

Here, we assessed the contribution of the fucosylated glycans sLe^X/A^ in cancer cell adhesion to E‐selectin, and the effects of fucosylation in cell signaling and proliferation in primary IDC. We observed that treatment with 2‐FF inhibited the adhesion of a human primary breast cancer cell line, CF1_T, to E‐selectin under physiologic flow conditions. Furthermore, treatment of CF1_T cells with 2‐FF significantly reduced their migration and proliferation rate, diminishing drastically the expression of cytokine growth factors and decreasing the activation of signal‐regulating protein kinases 1 and 2 (ERK1/2) and p38 mitogen‐activated protein kinase (MAPK). These findings provide mechanistic insights on the role of fucosylation in breast carcinogenesis, which suggests that inhibition of synthesis of fucosylated glycans may be beneficial in preventing tumor cell progression and metastasis.

## 2 Materials and methods

### 2.1 Reagents

Recombinant mouse E‐selectin/CD62E human immunoglobulin Fc chimera (E‐selectin‐Ig chimera, ‘E‐Ig’) was purchased from R&D Systems (Minneapolis, MN, USA). Anti‐CLA monoclonal antibody (mAb), clone HECA‐452, was from BioLegend (San Diego, CA, USA). Anti‐human CD29 mAb was from ImmunoTools (Friesoythe, Germany). Rat anti‐mouse CD62E, anti‐mouse immunoglobulins (Ig) mAb conjugated with horseradish peroxidase (HRP), and anti‐rat Ig conjugated with allophycocyanin (APC) mAbs were from BD Biosciences (San Jose, CA, USA). Anti‐rat IgG‐HRP, anti‐rat IgM‐HRP, and anti‐β‐tubulin mAbs were from Santa Cruz Biotechnology (Dallas, TX, USA). Anti‐human Ig‐fluorescein (FITC) mAb was from Sigma‐Aldrich (St. Louis, MO, USA). CellTrace™ 5,6‐carboxyfluorescein diacetate succinimidyl ester (CFSE) Cell Proliferation Kit was purchased from Molecular Probes (Leiden, Netherlands), Thermo Fisher Scientific (Waltham, MA, USA). Anti‐human cytokeratin, clone AE1/AE3, was from Dako (Santa Clara, CA USA). Anti‐p44/42 MAPK (ERK1/2), phospho‐ERK1/2 (P‐ERK), p38 MAPK, and phospho‐p38 (P‐p38) MAPK mAbs were from Cell Signaling Technology (Danvers, MA, USA).

### 2.2 Patients

This study involved patients from São José Hospital in Lisbon and from Cascais Hospital in Cascais, who underwent a mastectomy with gross resection of the primary tumor. The study was approved by the institutional research ethical committees, and all patient samples were obtained following informed consent.

### 2.3 Histological analysis

The immunohistochemical analysis of paraffin‐embedded tissue sections from IDC was performed as described below. Briefly, the slides were heated at 72 °C, deparaffinized, and hydrated. Endogenous peroxidase was blocked with 3% hydrogen peroxide and submitted to antigen recovery at 98 °C in citrate buffer pH 8. The slides were blocked with 5% BSA, incubated with E‐Ig chimera (5 μg·mL^−1^), followed by incubation with anti‐mouse CD62E mAb (1 : 50), and anti‐rat IgG‐HRP mAb (1 : 100) in tris‐buffered saline (TBS) supplemented with calcium. The specificity of the E‐Ig reactivity was always confirmed by adding EDTA (calcium chelator) to the TBS buffer, which always returned negative staining. The sLe^X/A^ expression was assessed using HECA‐452 mAb (1 : 50), followed by incubation with anti‐rat IgM‐HRP mAb (1 : 100) in TBS. The development was performed using Dako REAL DAB+ chromogen from Kit Dako REAL EnVision Detection System, Peroxidase/DAB. Nuclear contrast staining was performed with hematoxylin. After immunohistochemistry, slides were washed, dehydrated, treated with increasing concentrations of alcohol (75%, 90%, and 99%), cleared in xylene, and mounted with synthetic mounting medium (Quick‐D‐M‐Klinipath). The immunohistochemical staining was assessed by an experienced pathologist that was not informed of the status of slides. Immunohistochemical profile of breast tumor namely estrogen receptor, progesterone receptor, human epidermal growth factor receptor 2 (HER2), Ki‐67, and cytokeratin 5, was determined at the Cascais Hospital, and results were obtained from clinical pathology reports.

### 2.4 Primary cell culture

Part of the tumor tissue collected from patients was placed in growth medium [Dulbecco's modified Eagle's medium (DMEM) with 20% fetal bovine serum, glutamine, and antibiotics] immediately after surgical removal. Tumor tissue was minced and incubated in collagenase overnight to obtain a tumor cell suspension. Cells were then cultured in T‐25 flasks at 37 °C with 5% CO_2_. Medium was changed weekly, and cell cultures were trypsinized and passaged when sufficient growth colonies were noted. After 5–10 passages, the serum concentration was reduced to 10%, and the cells were then fed 2–3 times per week by replacement of the medium.

### 2.5 Immortalization of CF1 breast cancer primary cells

Cell immortalization of the CF1 primary cell line was performed by viral transduction of the human telomerase reverse transcriptase (hTERT) gene. hTERT is a catalytic subunit of the enzyme telomerase, which, together with the telomerase RNA component, comprises the most important unit of the telomerase complex, providing primary cells with the ability to achieve continuous cell proliferation in culture. Briefly, lentivirus containing *hTERT* was produced through the cotransfection of three different plasmids into the HEK293 cell line. Two plasmids coded for the viral capsid and the packaging proteins, integrase and reverse transcriptase, and a third plasmid had an insertion of the *hTERT* gene under a CMV promoter, together with a gene encoding resistance to blasticidin. *hTERT* gene was thus packaged inside a lentiviral particle that exited HEK293 cells into the culture medium. CF1 cells were cultured at 30% confluency, and lentivirus was added at a titer of 10^6^ viral particles·mL^−1^ together with 6 mg·mL^−1^ of polybrene (Sigma‐Aldrich). After 72 h of incubation in a 37 °C 5% CO_2_ atmosphere, blasticidin was added to kill nontransduced cells. *hTERT*‐transduced cells were thereafter named the ‘CF1_T cell line’.

### 2.6 Flow cytometry

The cell surface expression of sLe^X/A^ glycans was analyzed by flow cytometry using HECA‐452 mAb staining, followed by APC‐labeled secondary antibody. E‐Ig chimera staining was performed in the presence of PBS‐CaCl_2_ (Sigma‐Aldrich), followed by anti‐human Ig FITC. Specificity for E‐Ig binding was confirmed by control assays in PBS with 2 mm EDTA. Intracellular staining to detect the expression of cytokeratins, ERK1/2, P‐ERK, p38, and P‐p38 was performed using the Fixation/Permeabilization Solution Kit (BD Biosciences), before staining. Antibody staining was performed for 30 min at 4 °C followed by incubation with fluorescent‐labeled secondary antibodies. Background levels were determined in control assays by incubating cell suspensions with only fluorescent‐labeled secondary antibodies.

### 2.7 Confocal laser scanning microscopy

Cells were cultured on glass coverslips overnight and then fixed with 3.7% paraformaldehyde. After blocking with 1% BSA, cells were stained using E‐Ig chimera, followed by anti‐human Ig FITC in the presence of PBS‐CaCl_2_. After permeabilization with 0.1% Triton X‐100, F‐actin was stained with Alexa Fluor 568 phalloidin (Molecular Probes). Images were acquired with a Leica TCS SP2 AOBS confocal microscope. Representative cross‐sectional confocal images were selected after Z‐stacking.

### 2.8 2‐FF treatment

2‐FF was kindly provided by P. Senter from Seattle Genetics (Bothell, WA, USA). The CF1_T cell line was treated or not with 1 mm of 2‐FF inhibitor in DMEM with 10% fetal bovine serum, glutamine, and antibiotics.

### 2.9 SDS/PAGE and western blot

Cells were lysed in lysis buffer consisting of 150 mm NaCl, 2 mm CaCl_2_, 50 mm Tris (pH 7.4), 1 mm phenylmethylsulfonyl fluoride, 2% NP‐40, and 1 EDTA‐free protease inhibitor cocktail tablet (Roche, Indianapolis, IN, USA). Cell lysates were vortexed overnight at 4 °C, and supernatants were cleared by centrifugation for 10 min at 10 000 ***g***, then stored at −80 °C until use. Protein concentrations were determined using Pierce BCA protein assay kit (Thermo Scientific). Samples were electrophoresed in 8% SDS/PAGE. SDS/PAGE‐resolved proteins were transferred to nitrocellulose membranes (Bio‐Rad, Amadora, Portugal), and membranes were blocked with TBS/0.1% Tween‐20 with 10% dry milk for 1 h. In case of ERK and p38 staining, dry milk was replaced by 4% BSA. Immunoblots were stained with primary mAbs overnight at 4 °C and subsequently with appropriate HRP‐conjugated secondary antibodies for 1 h at room temperature. After rinsing with TBS/0.1% Tween‐20, Lumi‐light western blotting substrate (Roche) was used as developing reagent.

### 2.10 Gene expression analysis by real‐time PCR

RNA extraction was performed using the GenElute Mammalian Total RNA Purification kit (Sigma‐Aldrich), following the manufacturer's instructions. After DNase (NZYTech, Lisbon, Portugal) treatment, 1 μg of total RNA was reverse‐transcribed using the random primer‐based High Capacity cDNA Archive Kit (Applied Biosystems, Waltham, MA, USA) and the real‐time PCR was performed in a 7500 Fast Real‐Time PCR System with Master Mix, and TaqMan assays from Applied Biosystems. The assay IDs for each gene provided by the manufacturer were as follows: Hs00356857_m1 (fucosyltransferase 3—*FUT3*); Hs01106466_s1 (fucosyltransferase 4—*FUT4*); Hs00704908_s1 (fucosyltransferase 5—*FUT5*); Hs0326676_s1 (fucosyltransferase 6—*FUT6*); Hs00237083_m1 (fucosyltransferase 7—*FUT7*); Hs00276003_m1 (fucosyltransferase 9—*FUT9*); Hs00327091_m1 (fucosyltransferase 10—*FUT10*); Hs00543033_m1 (fucosyltransferase 11—*FUT11*); Hs00174097_m1 (interleukin 1 beta—*IL‐1*β); Hs00174131_m1 (interleukin 6—*IL‐6*); HS00174103_m1 (interleukin 8—*IL‐8*); Hs00171257_m1 (transforming growth factor beta1—*TGF‐*β*1*); Hs00170433_m1 (*HER2*); Hs00900055_m1 (vascular endothelial growth factor—*VEGFA*); Hs01052937_m1 (*VEGFR1* (*Flt‐1*)); Hs00911700_m1 (*VEGFR2* (*KDR*)); and Hs00960934_m1 (basic fibroblast growth factor—*FGF2*). The relative mRNA levels were normalized against the arithmetic mean of the endogenous control gene (β*‐ACTIN* and *GAPDH*) expression and calculated by the adapted formula 2^−ΔCt ^× 1000, which infers the number of mRNA molecules of the gene of interest per 1000 molecules of the endogenous controls (Videira *et al*., 2009). ΔCt stands for the difference between the cycle threshold of the target gene and that of the endogenous controls. Relative quantification (RQ) measures the relative change in mRNA expression levels from a given sample (2‐FF‐treated cells) relative to the reference sample (untreated cells) and was calculated using the formula RQ = 2−(ΔCtsample − ΔCtcontrol). The efficiency for each primer/probe was above 95%, as determined by the manufacturer.

### 2.11 Cell proliferation measurement

To study cell proliferative capacity, cells were labeled with CellTrace™ CFSE Cell Proliferation Kit (Molecular Probes) during 5 days of 2‐FF treatment. Cells were resuspended in DMEM at the final concentration of 1 × 10^6^ cells·mL^−1^ and incubated with 5 μm CFSE following the manufacturer's instructions. CFSE‐labeled cells were cultured for additional 9 days (total of 14 days after treatment). Cell fluorescence was measured using Attune Flow cytometer, and data were analyzed with modfit lt 3.2 software (Verity Software House, Topsham, ME). This analysis allowed quantification of the cell proliferation index, which represents the fold expansion of the overall culture (i.e., the average number of cells that were originated from a single cell of the parental generation). The parental generation was set based on the analysis of data obtained from the cells maintained at 24 h in culture after CFSE staining.

### 2.12 Analysis of cell motility using a wound‐healing assay

Cell motility was tested in a wound‐healing migration assay. CF1_T cells were seeded into 12‐well microplates and grown to confluence. A uniform scratch was made in the monolayer with a sterile 200‐μL pipette tip, and the suspended cells and debris were washed away with the addition of fresh medium. At 0 and 16 h after wounding, scratched regions were photographed with an inverted microscope equipped with a digital camera. The wounded area in the photographs was measured using imagej software (Schneider *et al.,* 2012), and the percentage of closed area was calculated using the formula: ((wounded area (24 h)/wounded area (0 h)) × 100) − 100.

### 2.13 Cell adhesion to E‐selectin using alternative Stamper–Woodruff assay

For the analysis of adherence of CF1_T cells to E‐selectin, adapted shear‐dependent Stamper–Woodruff assays were performed (Dimitroff *et al*., 2003). Briefly, glass slides were spotted with E‐Ig chimera, and the unoccupied slide area was blocked with 1% BSA. CF1_T cells treated or not with 1 mm 2‐FF for 5 days, or with sialidase [*Clostridium perfringens* (Roche Diagnostics) as described in Silva *et al*. (2016)], were resuspended in Hank's balanced salt solution (HBSS) containing 2 mm CaCl_2_ (HBSS‐Ca), overlaid onto the E‐Ig chimera spots in the glass slides, and incubated with orbital rotation at 80 r.p.m. for 30 min, at 4 °C. The slides were immersed in HBSS‐Ca to remove the nonadherent cells, and adherent cells were fixed with 3% glutaraldehyde. Negative control assays were performed with HBSS containing 5 mm EDTA. Cell adherence to E‐selectin was examined under light microscopy at 100× magnification, and representative photomicrographs were taken for analysis. The number of adherent cells in each photomicrograph was counted using imagej software.

### 2.14 Statistical analysis

Data were analyzed using graphpad prism 6 (GraphPad Software, La Jolla, CA USA). Data with two groups were analyzed by Student's paired *t*‐test if the samples have a Gaussian distribution and, if not, Wilcoxon signed‐rank test was used. Differences were considered statistically significant when *P* < 0.05 (*), *P* < 0.01 (**), and *P* < 0.005 (***).

## 3 Results

### 3.1 IDC expresses E‐selectin ligands

**Figure 1:**
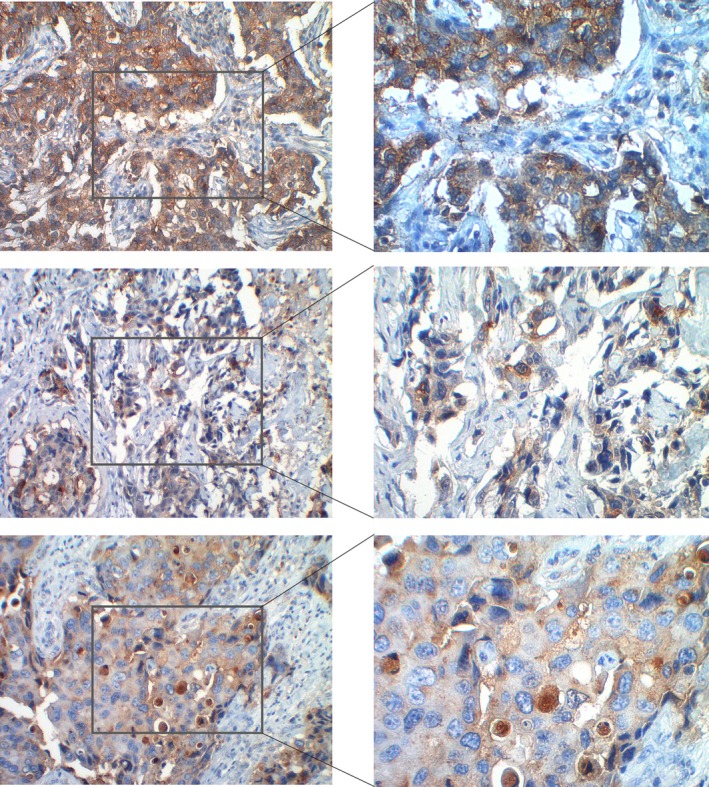
E‐selectin ligands are expressed by IDC. E‐selectin ligands were stained using E‐selectin Ig chimera (brown) by immunohistochemistry, as described in the 2 section. Representative photomicrographs of immunohistochemical analysis of three cases of IDC are shown at 200× (left) and 400× (right) magnification.

The expression of E‐selectin ligands in breast IDC was assessed by immunohistochemistry using E‐Ig chimera as a probe in paraffin‐embedded tissue sections. As shown in Fig. 1, IDC tissues exhibit expression of E‐selectin ligands. The E‐Ig reactivity was localized both in the plasma membrane and in the cytoplasmic structures with a remarkable reactivity in poorly differentiated areas. When comparing breast IDC with different grades of differentiation, grade 3 (poorly differentiated) IDC showed a higher number of cases positive for E‐selectin ligands than the grade 1 (well‐differentiated) IDC (Table 1). Negative control slides using buffer supplemented with EDTA do not show E‐Ig staining (data not shown), confirming the specificity of the immunohistochemical analysis. The expression of sLe^X/A^ was also detected in the different grades of IDC (Table 1).

**Table 1:** E‐selectin ligands and sLe^X/A^ expression in IDC tissues analyzed by immunohistochemistry

	E‐selectin ligand expression (E‐Ig staining)		sLe^X/A^ expression (HECA‐452 staining)	
	Total number of cases, *n* = 15		Total number of cases, *n* = 9	
	Positive	Negative	Positive	Negative
Grade 1	1	5	1	2
Grade 2	1	2	1	2
Grade 3	5	1	2	1

Paraffin‐embedded sections were stained with E‐Ig chimera (*n* = 15) or the HECA‐452 mAb (*n* = 9) to assess the expression of E‐selectin ligands and the expression of sLe^X/A^.

### 3.2 Characterization of primary cell lines from invasive ductal breast carcinoma patients

**Figure 2:**
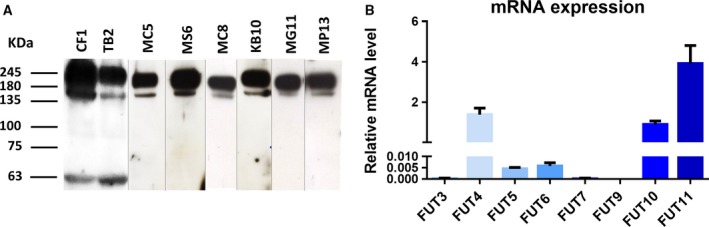
(A) Western blot analysis of the sialofucosylated glycoproteins expressed in the established primary breast cancer cell cultures. Whole‐cell lysate of the primary cell lines was resolved by SDS/PAGE electrophoresis and immunoblotted with HECA‐452 mAb, which recognizes sLe^X^ ^/A^ determinants. Two major HECA‐452‐reactive bands were observed for all the primary cell cultures. Blot images obtained from different gels. (B) Gene expression of α1,3/4‐FUTs in CF1 primary breast cancer cells analyzed by RT‐PCR. Values of relative mRNA level correspond to the amount of RNA copies of each FUT α1,3/4‐FUTs (FUT3, FUT4, FUT5, FUT6, FUT7, FUT9, FUT10, FUT11) per each 1000 RNA copies of housekeeping genes (β*‐ACTIN* and *GAPDH*).

To better understand the role of E‐selectin ligands in IDC pathogenesis, a diverse group of primary cells with a broad expression of breast cancer standard markers, such as estrogen receptor, progesterone receptor, HER2, Ki67, and cytokeratin 5, was established (Table 2). The epithelial origin of these cells was confirmed by the expression of several cytokeratin proteins to exclude mesenchymal cell contamination (see Fig. S1). Isolated primary cells duplicated between 3 and 15 days, and all cultures grew until passage 10, except for CF1 cells that grew until passage 30 before entering senescence. To specifically assess the expression of sLe^X^ and sLe^A^ determinants, cells were stained with HECA‐452 mAb. By flow cytometry, all primary cells showed HECA‐452 reactivity, with the CF1 cells exhibiting the highest expression (Table 3). Western blot analysis revealed that all primary cells were stained for two major HECA‐452‐reactive bands at ~ 200–250 kDa and ~ 150 kDa (Fig. 2A). Regarding the expression of α‐1,3/4‐FUTs, all cells showed the same profile, expressing FUT4, ‐5, ‐6, ‐10, and ‐11, and not expressing FUT3, ‐7, and ‐9 (Fig. 2B). To perform further functional studies, we immortalized the CF1 cell line by *hTERT* transduction. The resultant CF1_T immortalized cells continued to express sLe^X/A^ determinants, although with lower levels than the original, it maintained with the same pattern of E‐selectin‐reactive glycoproteins and FUT expression (Fig. S2). Accordingly, to perform the subsequent studies, we used this immortalized CF1_T cell line as representative of CF1 primary cells.

**Table 2:** Clinical information about breast cancer features from tissues used to establish primary cell cultures. na. not analyzed

Cancer cell culture	Patient's age	Histologic type	Clinical stage	Tumor markers				
				Estrogen receptor (%)	Progesterone receptor (%)	HER2	Ki.67 (%)	Cytokeratin 5
CF1	31	IDC	Stage IIA (T2, N0, M0)	25	15	+	5	Negative
TB2	41	IDC	Stage IIA (T1c, N1, M0)	100	90	−	20	na
MC5	61	IDC	Stage IA (pT1c, pN0 (sn)(i‐))	80	40	+	10	Negative
MS6	74	IDC	Stage IA (pT1c, pN0 (sn)(i‐))	100	60	−	10	Negative
MC8	62	IDC	Stage IIB (pT2, pN1a (1/16))	100	50	−	10	Negative
KB10	62	IDC	Stage IA (pT1c, pN0 (sn)(i‐))	100	20	−	5	Negative
MG11	47	IDC	Stage IA (pT1c, pN0 (sn)(i‐))	90	80	−	20	Negative
MP13	73	IDC	Stage IA (pT1b, pN0 (sn)(i‐))	90	0	−	10	Negative

**Table 3:** E‐selectin ligand expression on primary breast cancer cells analyzed by flow cytometry. + → MFI < 1000; ++ → 1000 < MFI < 5000; +++ → 5000 < MFI < 7500; ++++ → MFI > 7500

	CF1	TB2	MC5	MS6	MC8	KB10	MG11	MP13
HECA‐452 staining	++++	++	+	+++	++	++	+	+++

### 3.3 2‐FF treatment in CF1_T cells abrogates their capacity to bind E‐selectin

**Figure 3:**
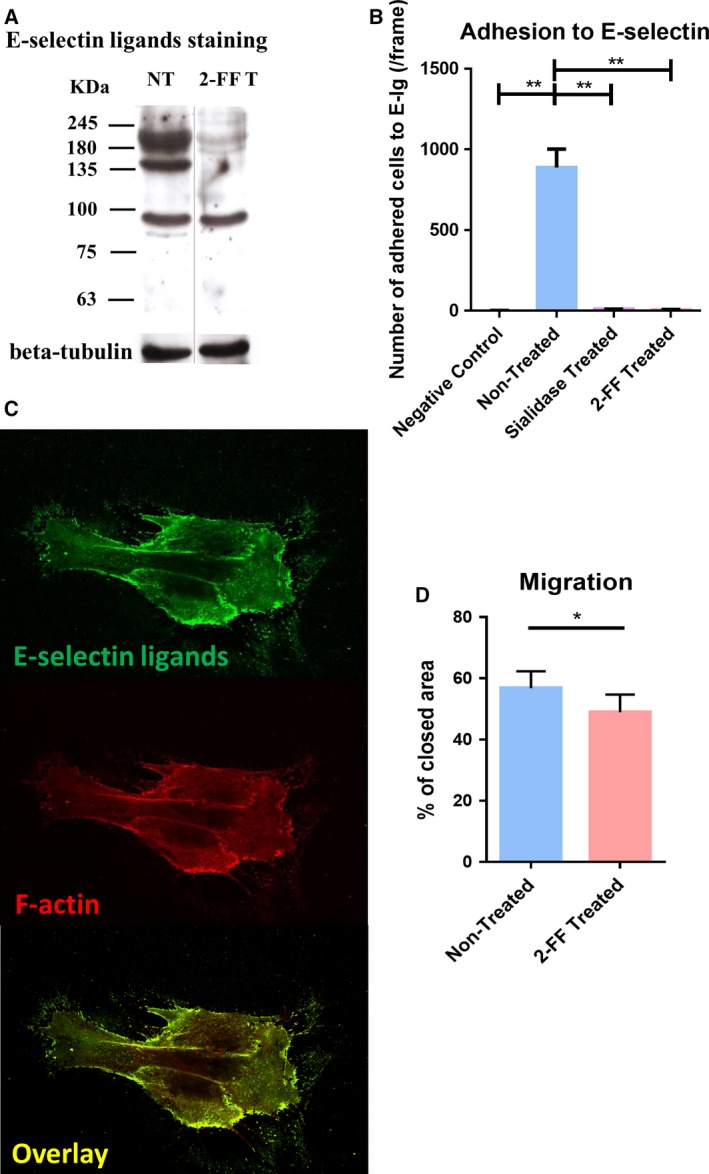
CF1_T cell line treated with 2‐FF compound loses functional E‐selectin ligands and exhibits a reduced cell migration capacity. (A) Effect of 2‐FF on the expression of sialofucosylated glycoproteins by CF1_T cells. The CF1_T cell line was treated with 1 mm of 2‐FF inhibitor (2‐FF T) or not (NT), for 5 days. Total lysate proteins were stained with the HECA‐452 mAb and analyzed by western blot. Βeta‐tubulin expression was used as loading control. (B) Effect of 2‐FF and sialidase on the capacity of CF1_T cells to adhere to E‐selectin under flow conditions. The CF1_T cells were treated or not with 2‐FF for 5 days, or sialidase for 1 h, and their capacity to adhere to E‐Ig chimera was analyzed under flow conditions by an alternative Stamper–Woodruff assay. Cells were added in calcium buffer over an E‐Ig spot and incubated with an orbital rotation at 80 r.p.m. for 30 min, at 4 °C. Assays performed in EDTA buffer were used as negative control. [*n* = 4; *P* < 0.01 (**)] (C) Cellular distribution of E‐selectin ligands in CF1_T cells. Cells were stained with E‐Ig chimera plus anti‐human IgG‐FITC (top image) and Alexa Fluor 568 phalloidin (middle image) and analyzed by confocal microscopy. Overlay of both images is displayed on the bottom. (D) The migratory ability of CF1_T cells after 10 days of 2‐FF treatment was analyzed by scratch wound‐healing assay [*n* = 5; *P* < 0.05 (*)].

To assess whether inhibition of fucosylation would have an effect on the ability of CF1_T cell line to bind E‐selectin, we treated these cells with the fucosylation inhibitor, 2‐FF (Okeley *et al*., 2013). 2‐FF did not affect cell viability (Fig. S3). By western blot analysis, we observed that 2‐FF‐treated CF1_T cells expressed significantly less E‐selectin ligands in comparison with nontreated cells (Fig. 3A). Similarly, by flow cytometry, we observed the downregulation of E‐selectin ligands and sLe^X/A^ expression in 2‐FF‐treated cells (Fig. S4). 2‐FF‐treated CF1_T cells completely lose their ability to bind to E‐selectin under flow conditions, as assessed by a modified Stamper–Woodruff assay. Similarly, the treatment with sialidase, which removes cell surface sialic acid, also prevents the binding of CF1_T cells to E‐selectin (Fig. 3B). The effect of 2‐FF compound on the ability of cells to adhere to E‐selectin was also confirmed in the TB2 primary breast IDC cells (Table 2), which retrieved the same results as with CF1_T cell line (Fig. S5). These data show the importance of sialofucosylated structures, such as sLe^X/A^, expressed by CF1_T cells in mediating E‐selectin adhesion.

### 3.4 2‐FF‐treated CF1_T cells have lower migration capacity than nontreated cells

In metastatic breast cancer cell lines, previous studies have shown that E‐selectin ligands are codistributed with F‐actin; that is, both are distributed in similar locations within the cells (Zen *et al*., 2008). Here, to assess the distribution of E‐selectin ligands in the CF1_T cells, we performed a costaining of E‐selectin ligands (using E‐Ig chimera) and F‐actin (phalloidin staining). Notably, our data showed that E‐selectin ligands are mainly localized at the leading edge of CF1_T cells, with a strong codistribution with F‐actin (Fig. 3C), suggesting that E‐selectin ligands play a role in cell motility and migration. To better understand this role, we treated the cells with the 2‐FF inhibitor to abrogate E‐selectin ligand expression in CF1_T cells and then evaluated their migration capacity in a wound‐healing assay. We verified that 16 h after wound, 2‐FF‐treated CF1_T cells sealed 49% of the opened ‘wound’, which was significantly reduced compared with nontreated cells, which covered 57% of the region (Fig. 3D). Our results indicate a role of fucosylation in CF1_T cell migration.

### 3.5 Treatment with 2‐FF reduces cell proliferation and expression of growth factors

**Figure 4:**
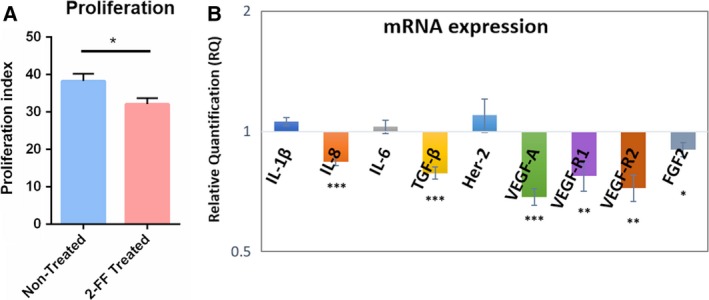
2‐FF treatment on CF1_T cells induces lower expression of growth factors and reduced cell proliferation rate. (A) Effect of 2‐FF on CF1_T cell proliferation. CF1_T cells were treated, or not, with 2‐FF, for a total of 14 days. At day 5 of treatment, the cells were stained with CFSE dye and then analyzed after another 9 days of treatment (total of 14 days). CFSE dilution was analyzed by flow cytometry and the proliferation index calculated as the fold expansion of the overall culture using the modfit software [*n* = 8; *P* < 0.05 (*)]. (B) The gene expression of growth factors and cytokines on 2‐FF‐treated CF1_T cell line was compared to nontreated CF1_T cell line (ratio 1). RQ indicates the relative change in mRNA expression levels from the 2‐FF‐treated cells relative to the untreated cells [*n* = 6; *P* < 0.05 (*), *P* < 0.01 (**), and *P* < 0.005 (***)].

The suppression of FUT expression in cancer has been shown to be able to attenuate their cell proliferation (Kawai *et al*., 2013). To assess the influence of fucosylation abrogation in cell proliferation, we used the CFSE dilution method as a readout to measure proliferation indexes. Briefly, CF1_T cells were treated with 2‐FF compound, and at day 5, they were stained with CFSE and analyzed by flow cytometry after 9 more days (14 days of 2‐FF treatment). The data showed that the proliferation index of nontreated CF1_T cells was statistically higher than the index of 2‐FF‐treated cells (Fig. 4A), suggesting a role of fucosylated determinants in CF1_T cell proliferation. As the expression of several growth factors and pro‐inflammatory cytokines is associated with increased proliferation, migration, invasion, and survival in cancer, we then evaluated the effect of 2‐FF on the expression of representative growth factors or their receptors and cytokines in CF1_T cells. As represented in Fig. 4B, 2‐FF‐treated CF1_T cells express statistically significant decreases in FGF2 (RQ = 0.9002), VEGFA (RQ = 0.6859), VEGFR1 (RQ = 0.7756), VEGFR2 (RQ = 0.7228), TGF‐β (RQ = 0.7871), and IL‐8 (RQ = 0.8412) than in nontreated CF1_T cells (Fig. 4B). These results indicate that 2‐FF decreases the expression of relevant growth factors and their receptors, which may underlie the observed effect of 2‐FF treatment on cell proliferation and migration previously reported.

### 3.6 Suppression of MAPK signaling pathways by 2‐FF treatment

**Figure 5:**
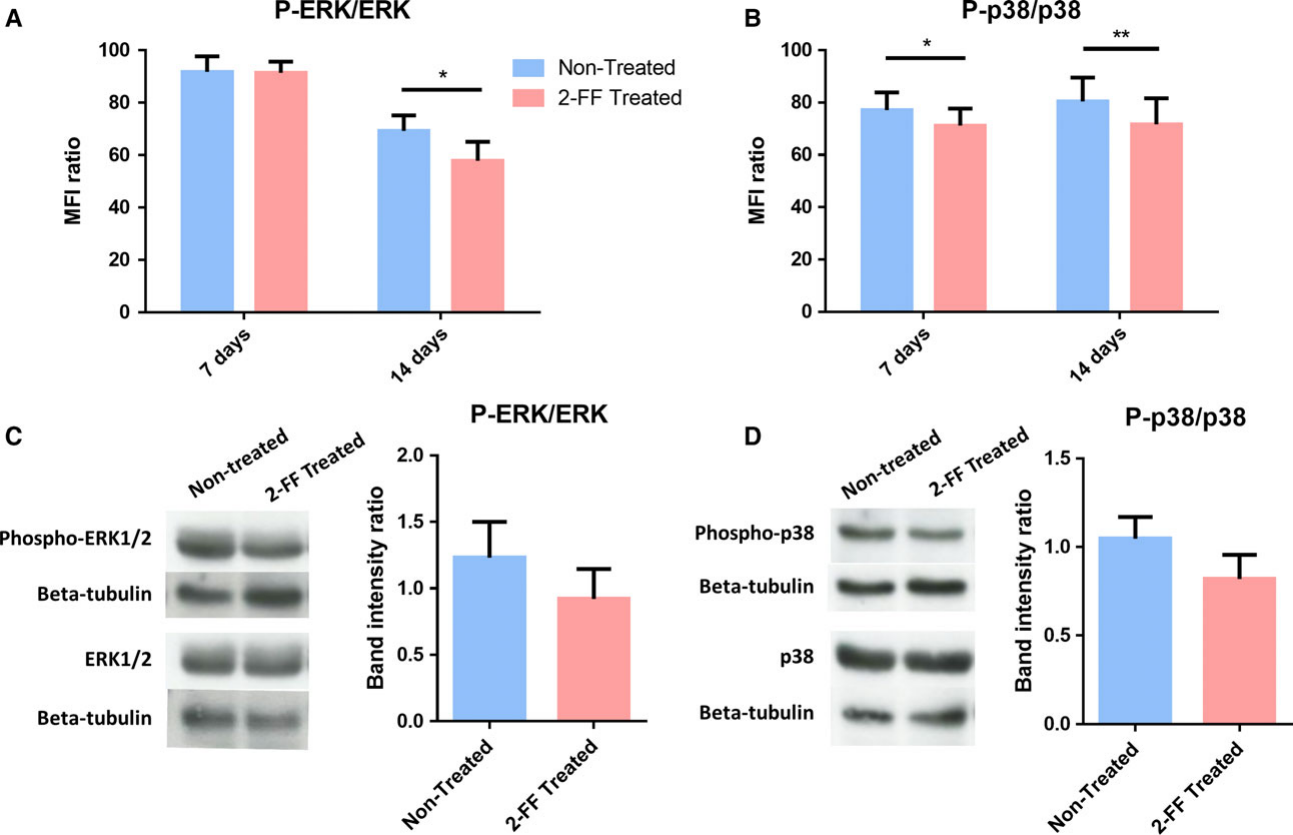
ERK1/2 and p38 MAPK pathways are downregulated by 2‐FF treatment of CF1_T cell line. (A,B) CF1_T cell line treated with 2‐FF for 7 and 14 days was analyzed regarding their expression of P‐ERK, total ERK1/2 (ERK), P‐p38, and total p38 (p38) kinases by flow cytometry. Ratios between P‐ERK and ERK expression ((P‐ERK/ERK) × 100) and P‐p38 and p38 expression ((P‐p38/p38) × 100) are represented in the graphs A and B, respectively [*n* = 4; *P* < 0.05 (*), *P* < 0.01 (**)]. (C,D) CF1_T cell line was treated with 2‐FF inhibitor for 14 days. Total lysate proteins were stained with anti‐P‐ERK1/2, anti‐ERK1/2, anti‐P‐p38, or anti‐p38 mAb and analyzed by western blot. Βeta‐tubulin expression was analyzed as a loading control. Band intensity was measured in imagej software, and P‐ERK/ERK ratio (C) and P‐p38/p38 ratio (D), after normalization with loading control, are represented in the graphs (*n* = 4).

As the activation of ERK1/2 and the p38 MAPK is involved in the proliferation and migration of breast cancer cells (Chen *et al*., 2009; Zhou *et al*., 2008), we then assessed the effect of the 2‐FF treatment in the MAPK signaling pathways. The ratio of P‐ERK/ERK1/2 to P‐p38/p38 expression was assessed by intracellular staining by flow cytometry, upon 7 and 14 days of 2‐FF treatment. Interestingly, after 14 days of treatment, a significantly reduced expression of P‐ERK on cells treated with 2‐FF comparing with the nontreated cells was observed [mean fluorescence intensity (MFI) ratio = 57.88 ± 7.24 to 69.30 ± 5.94, respectively]. No differences were observed after 7 days of treatment (MFI ratio = 91.46 ± 4.27 to 91.81 ± 5.86; Fig. 5A). After 7 and 14 days of treatment, we detected a significantly lower expression of P‐p38 in 2‐FF‐treated cells than in nontreated cells (MFI ratio = 71.20 ± 6.49 to 77.09 ± 6.77, for 7 days, and 71.68 ± 9.91 to 80.42 ± 9.16, for 14 days of treatment, respectively; Fig. 5B). Similar results were obtained by western blot, as 2‐FF treatment downregulates the expression of P‐ERK and of P‐p38 in comparison with nontreated cells (band intensity ratio = 0.92 ± 0.22 to 1.23 ± 0.27, for ERK1/2 pathway, and 0.82 ± 0.14 to 1.05 ± 0.12, for p38 pathway, respectively; Fig. 5C,D). Overall, these data suggest that fucosylated structures contribute to cell proliferation and migration through ERK1/2 and p38 MAPK pathways in breast cancer cells.

## 4 Discussion

Breast cancer cells are reported to overexpress the fucosylated sLe^X^ and sLe^A^ antigens, which correlates with metastasis (Jeschke *et al*., 2005; Renkonen *et al*., 1997; Wei *et al*., 2010). In this work, we aimed to understand the role of these determinants and of fucosylation in general, in the pathobiology of IDC, the most common type of breast cancer (Viale, 2012). As sLe^X^ and sLe^A^ are the prototypes of selectin ligands, we first evaluated E‐selectin binding by immunohistochemical staining of IDC tissue sections. E‐selectin ligands were found on both the cell cytoplasm and the plasma membrane, being more evident in poorly differentiated (grade 3) IDC tissue. Primary cancer cell cultures were then established from eight different IDC patient tissues, at different clinical stages of disease and different phenotypes. As expected, all the established primary breast cancer cell cultures expressed E‐selectin ligands. Furthermore, all cells exhibited the same profile of sLe^X/A^‐bearing glycoproteins, suggesting that IDC has a defined group of scaffold proteins decorated with sLe^X/A^. In other reports, the main α1,3/4‐FUTs associated with sLe^X^ biosynthesis in breast cancer included FUT3, FUT6, and FUT7 (Ding and Zheng, 2004; Julien *et al*., 2011; Matsuura *et al*., 1998). In our studies, we found that primary breast IDC cells do not express FUT3 or FUT7 and express FUT 5 and FUT6. FUT6 functions solely as an α1,3‐FUT, but FUT5 is both an α1,3‐ and α1,4‐FUT (Sackstein, 2009). Thus, our findings suggest that, in breast IDC, both FUT6 and FUT5 are involved in sLe^X/A^ biosynthesis. In the future, it would be relevant to assess the alteration of specific FUT gene expression in IDC cells, to confirm the role of FUT5 and FUT6.

To study the effect of fucosylation in IDC pathogenesis, we immortalized the CF1 cells (which display the highest levels of E‐selectin ligands) by transferring exogenous hTERT, allowing us to grow significant numbers of cells to continue this study. The hTERT immortalization enables extension of life span without altering the characteristic phenotypic properties of the cancer cells (Hooijberg *et al*., 2000; Ouellette *et al*., 2000). In agreement, the resultant CF1_T cell line still expresses E‐selectin ligands, FUTs, and sLe^X/A^—glycoproteins as the original cells. Nevertheless, it is reasonable to accept some caveats in our experimental model, generated by the genetic manipulation of the cells.

Expression of E‐selectin ligands, such as sLe^X^, has been positively associated with cell migration and motility in several cancer types (Pérez‐Garay *et al*., 2010; Radhakrishnan *et al*., 2011). Consistent with data obtained with MDA‐MB‐231 breast cancer cell line (Zen *et al*., 2008), the expression of E‐selectin ligands in CF1_T cell line is also localized in the leading edge of cells, associated with F‐actin, indicating a role of these ligands in cell migration. In fact, 2‐FF‐treated CF1_T cells, which did not express functional E‐selectin ligands, have significantly less migratory ability, supporting a role of sLe^X/A^ glycans and/or other fucosylated structures in cancer cell migration.

In further assays, we used the 2‐FF compound, a fucosylation inhibitor (Okeley *et al*., 2013), in CF1_T cells, to assess the role of fucosylation in the malignant features of these cells. The 2‐FF treatment leaded to a reduction in sLe^X/A^ cell surface expression to one‐fourth of the original level, which is in agreement with the original reports (Okeley *et al*., 2013). These results are also similar to studies where α1,3/4‐FUT expression or activity was suppressed (Hiraiwa *et al*., 1996; Shinoda *et al*., 1998; Trinchera *et al*., 2011), leading to significant reduction in sLe^X/A^ glycan expression and cell ability to bind E‐selectin under hemodynamic shear conditions. Nonetheless, it should not be excluded the possibility that 2‐FF may inhibit other fucosylations such as the core fucosylation mediated by the α1,6‐FUT8, also implicated in cell malignancy (Cheng *et al*., 2016). Interestingly, it was recently reported that in leukocytes, α1,3‐fucosylated structures, distinct from the sLe^X^, dampen cell trafficking by inhibiting the chemokine receptor CXCR2‐mediated signaling pathways (Buffone *et al*., 2017). The 2‐FF may also influence the expression of other glycans whose biosynthetic pathways have common steps with fucosylation. For instance, sialyltransferases may recognize the same acceptor substrate as FUTs, and it has been reported that knockout of FUTs involved in sLe^X^ biosynthesis results in an increase in sialylation (Noro *et al*., 2015). Interrupting fucosylation has the potential to disrupt other pathways in IDC by sLe^X^‐independent or selectin‐independent mechanisms.

Our results show that 2‐FF‐treated cells have a markedly reduced sLe^X^ content and exhibited a significantly reduced proliferation index. In agreement, it was shown that cancer cells with higher expression of sLe^X^ have a higher cell proliferation rate (Yusa *et al*., 2010). Remarkably, the expression of IL‐8, TGF‐β, VEGFA, VEGFR1, VEGFR2, and FGF2, which had been described as promoting cancer cell proliferation (Hsu *et al*., 1994; de Jong *et al*., 1998; Kajdaniuk *et al*., 2013; Liang *et al*., 2006; Ning *et al*., 2013; Sharpe *et al*., 2011), is significantly reduced on 2‐FF‐treated cells, suggesting that the decreased growth factors are the underlying mechanism for the reduced cell proliferation. The reduced induction of growth factor can, in turn, be attributable to altered glycosylation of the growth factors or their receptors, as it is the case of fucosylation of TGF‐β receptors that has been described as affecting the phosphorylation of the downstream molecules (Hirakawa *et al*., 2014). Nevertheless, while expression of growth factors is known to be associated with improved invasion and metastasis formation in breast cancer (Ning *et al*., 2013; Thielemann *et al*., 2013; Yao *et al*., 2007), this is the first study showing that the reduction in fucosylated structures (such as sLe^X/A^) is associated with lowering the expression of growth factors and receptors.

The regulation of cell proliferation is a complex process, being primarily regulated by external growth factors that activate different MAPK pathways. Taking this into account, we assessed the influence of 2‐FF treatment on the activation of the two main MAPK pathways, ERK1/2 and p38 pathways, which have been described to be involved in breast cancer cell proliferation and migration (Chen *et al*., 2009; Zhang and Liu, 2002; Zhou *et al*., 2008). We observed that 2‐FF treatment of CF1_T cells leads to a diminished activation/phosphorylation of ERK1/2 and p38 kinases. Among the affected growth factors, FGF2 has already been reported to be associated with ERK1/2 activation in breast cancer (Sharpe *et al*., 2011). Similarly, TGF‐β can also play a role in the activation of ERK1/2 and p38 MAPK during breast cancer cell proliferation (Galliher and Schiemann, 2007; Gomes *et al*., 2012). Thus, the low expression of growth factors could be one of the causes for the reduced activation of MAPK pathways observed in cancer cells with decreased fucosylation. These data strongly indicate that in IDC, which show a high expression of sLe^X/A^, the inhibition of fucosylation reduces E‐selectin ligand expression, cell proliferation, ERK1/2 and p38 MAPK activation, and growth factor expression.

As the 2‐FF is a universal inhibitor of fucosylation, in addition to the 2‐FF effect in decreasing sLe^X/A^ expression, we cannot exclude the effect on other fucosylated glycans or even other glycans whose biosynthesis shares a common substrate. Further studies are therefore necessary to investigate the potential contribution of each glycan and to fully elucidate the mechanisms underlying the altered biological functions.

## 5 Conclusion

Taken together, our data indicate that inhibition of fucosylation in primary breast IDC cells abrogates the expression of functional E‐selectin ligands, decreases the expression of growth factors, and negatively influences the activation of ERK1/2 and p38 pathways, leading to a reduced cell proliferation and migration. The increased understanding of fucosylation as a mediator of tumor cell adhesion and proliferation provides compelling logic to explore fucosylation‐directed therapeutic interventions as a means to combat IDC progression.

## Author contributions

MAC and PAV conceived and designed the project and wrote the manuscript. MAC performed main experiments. MS helped to conduct the experiments. CP and MM performed IHC staining of clinical samples, and JSR helped to conduct the immortalization process. IS collected tissue samples for primary culture establishment. MS, MJO, and RS provided guidance and assistance. All of the authors reviewed the manuscript before submission and approved the final manuscript.

## Supporting information


**Fig S1**. All CF1 primary cells express cytokeratins confirming their epithelial origin.

**Fig S2.** CF1_T cells continue to express E‐selectin ligands and α1,3/4‐FUTs after the immortalization process.

**Fig S3**. Treatment with 2‐FF does not induce cell death of CF1_T cell line.

**Fig S4**. CF1_T cell line treated with 2‐FF compound loses the expression of E‐selectin ligands and sLe^X/A^ glycans.

**Fig S5**. TB2 primary breast IDC cells treated with 2‐FF compound loses functional E‐selectin ligands.
