# Supplementary figures and images for: Inhibition of fucosylation in human invasive ductal carcinoma reduces E‐selectin ligand expression, cell proliferation, and ERK1/2 and p38 MAPK activation
Source: Mol Oncol. 2018 Mar 30;12(5):579–93. doi: 10.1002/1878-0261.12163 (PMC5928367; doi:10.1002/1878-0261.12163)

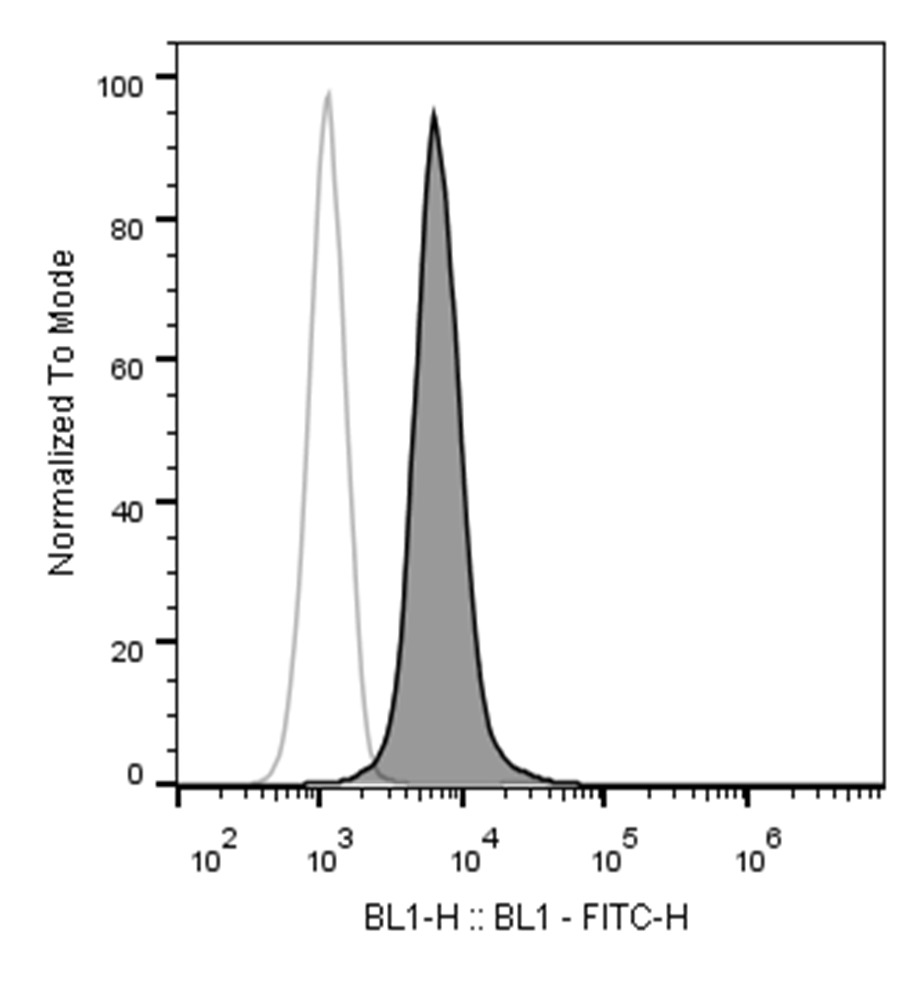

Supplement: Supplementary file 1 — Fig S1. All CF1 primary cells express cytokeratins confirming their epithelial origin. [file MOL2-12-579-s001.tif]

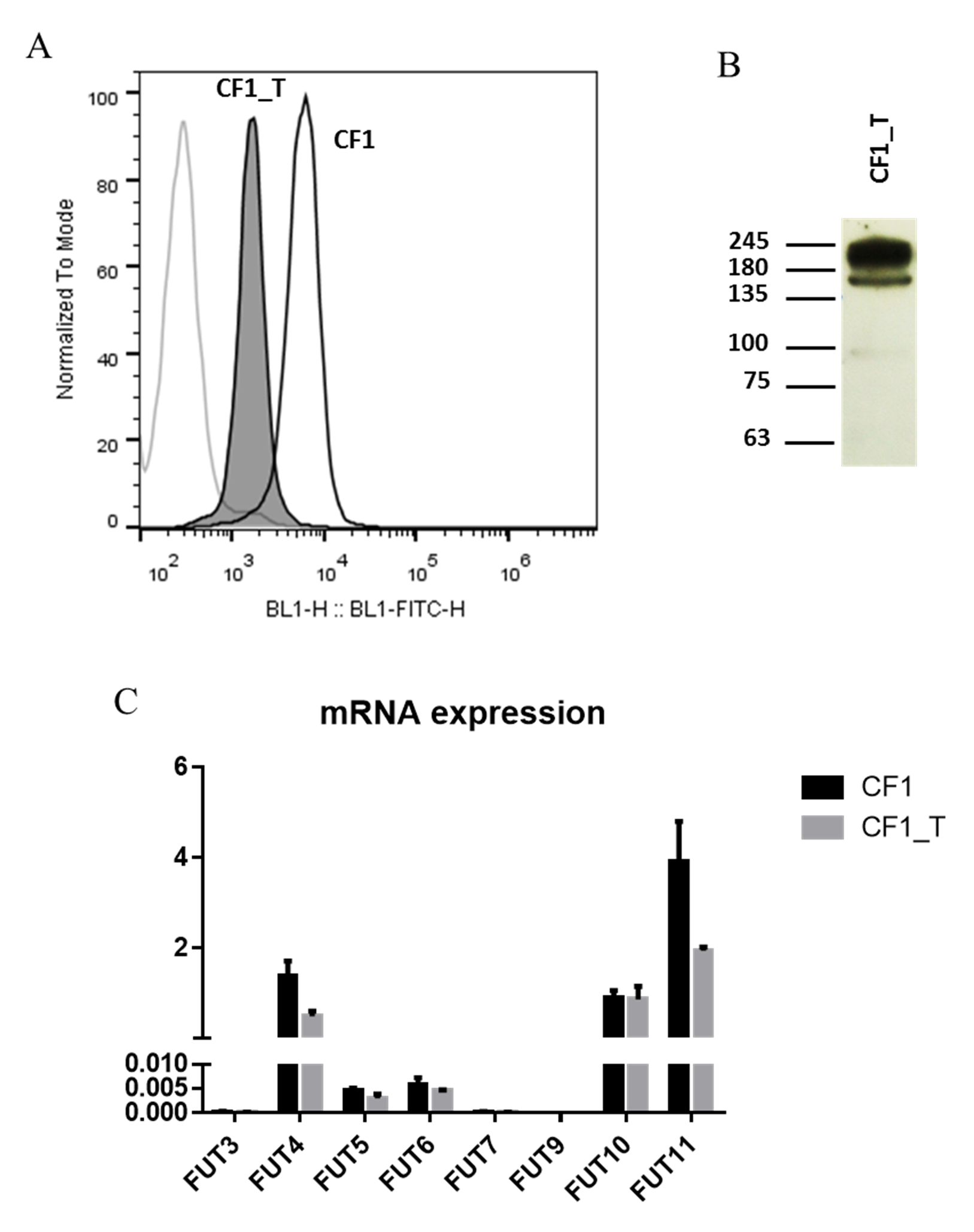

Supplement: Supplementary file 2 — Fig S2. CF1_T cells continue to express E‐selectin ligands and α1,3/4‐FUTs after the immortalization process. [file MOL2-12-579-s002.tif]

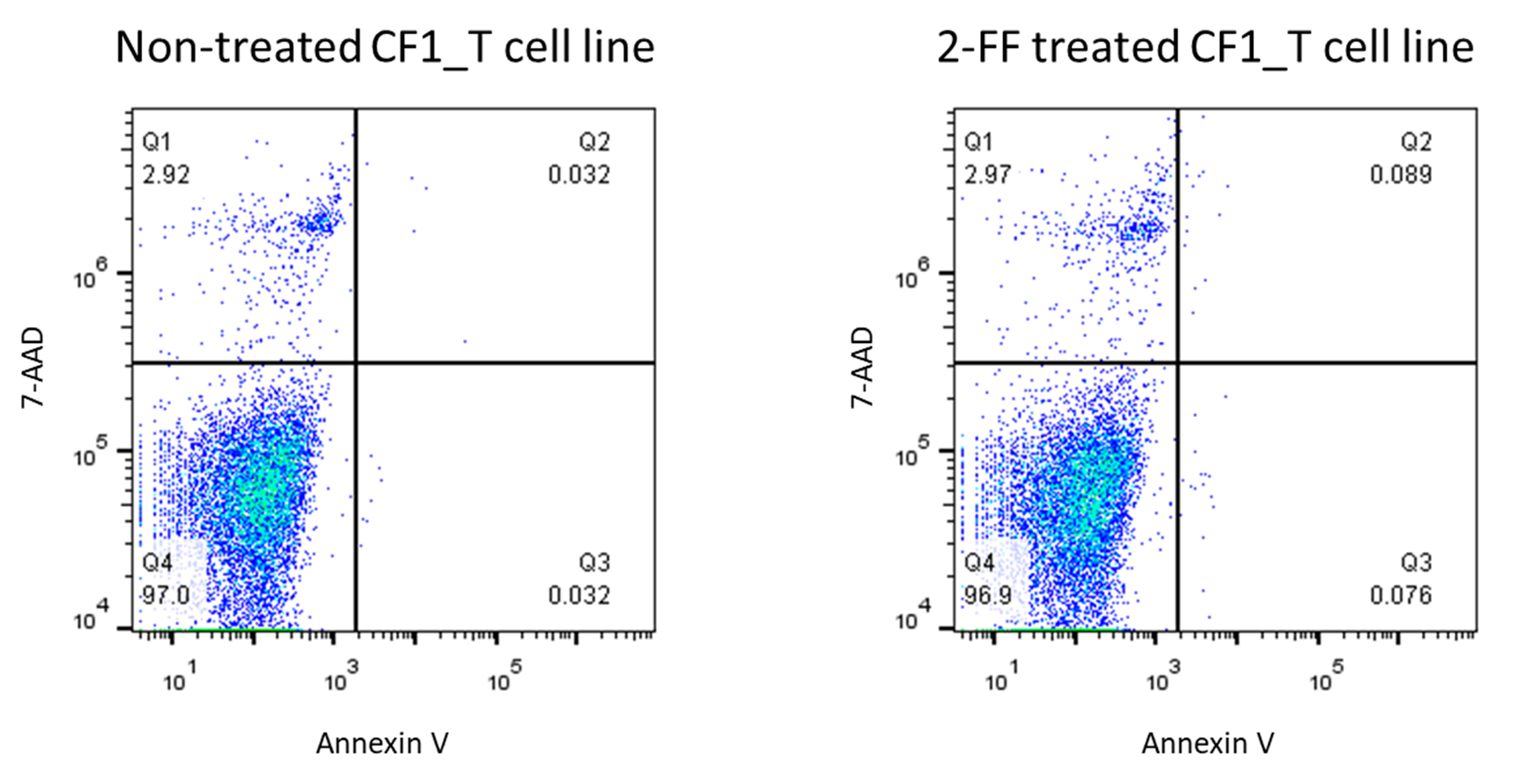

Supplement: Supplementary file 3 — Fig S3. Treatment with 2‐FF does not induce cell death of CF1_T cell line. [file MOL2-12-579-s003.tif]

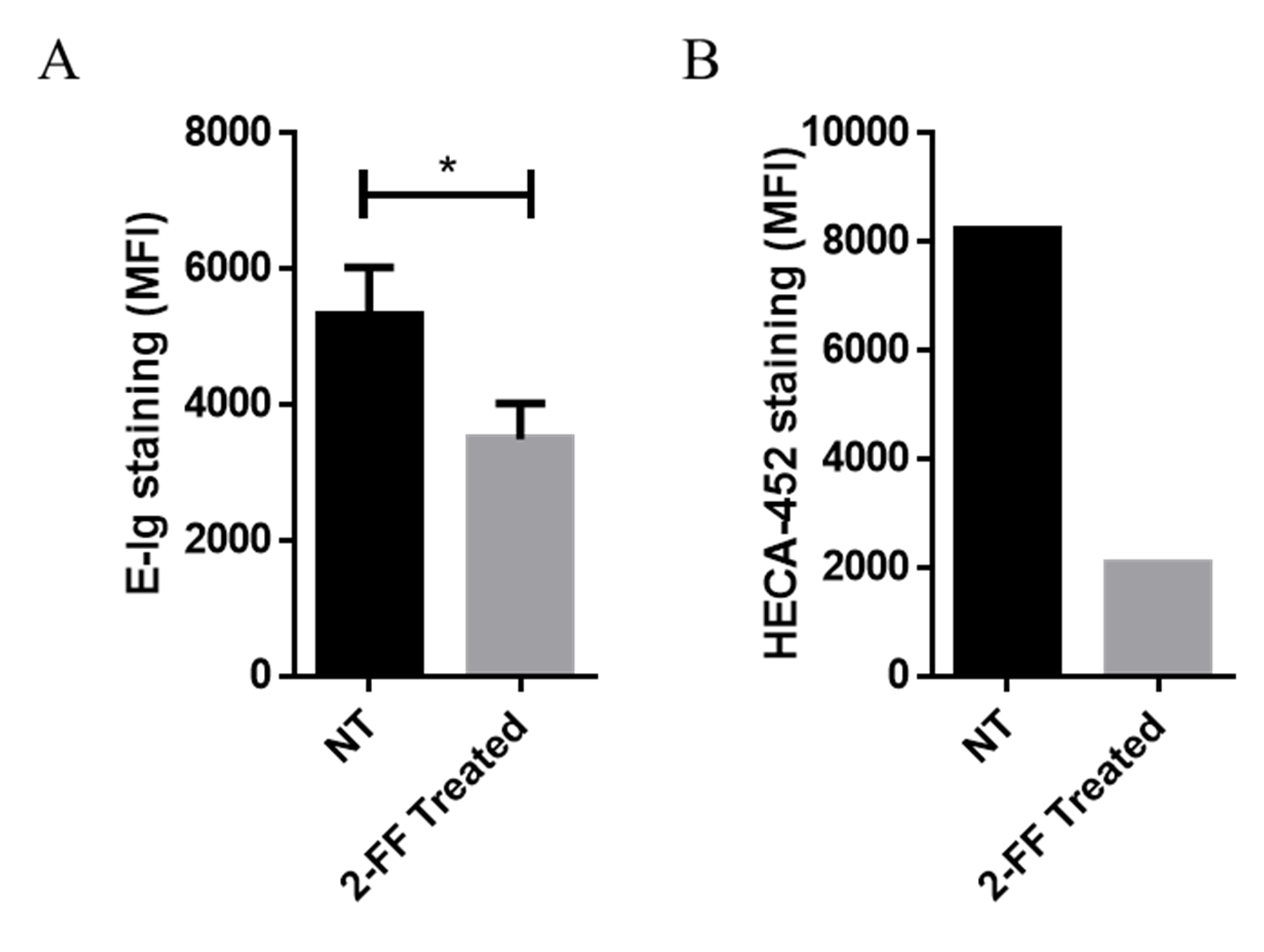

Supplement: Supplementary file 4 — Fig S4. CF1_T cell line treated with 2‐FF compound loses the expression of E‐selectin ligands and sLeX/A glycans. [file MOL2-12-579-s004.tif]

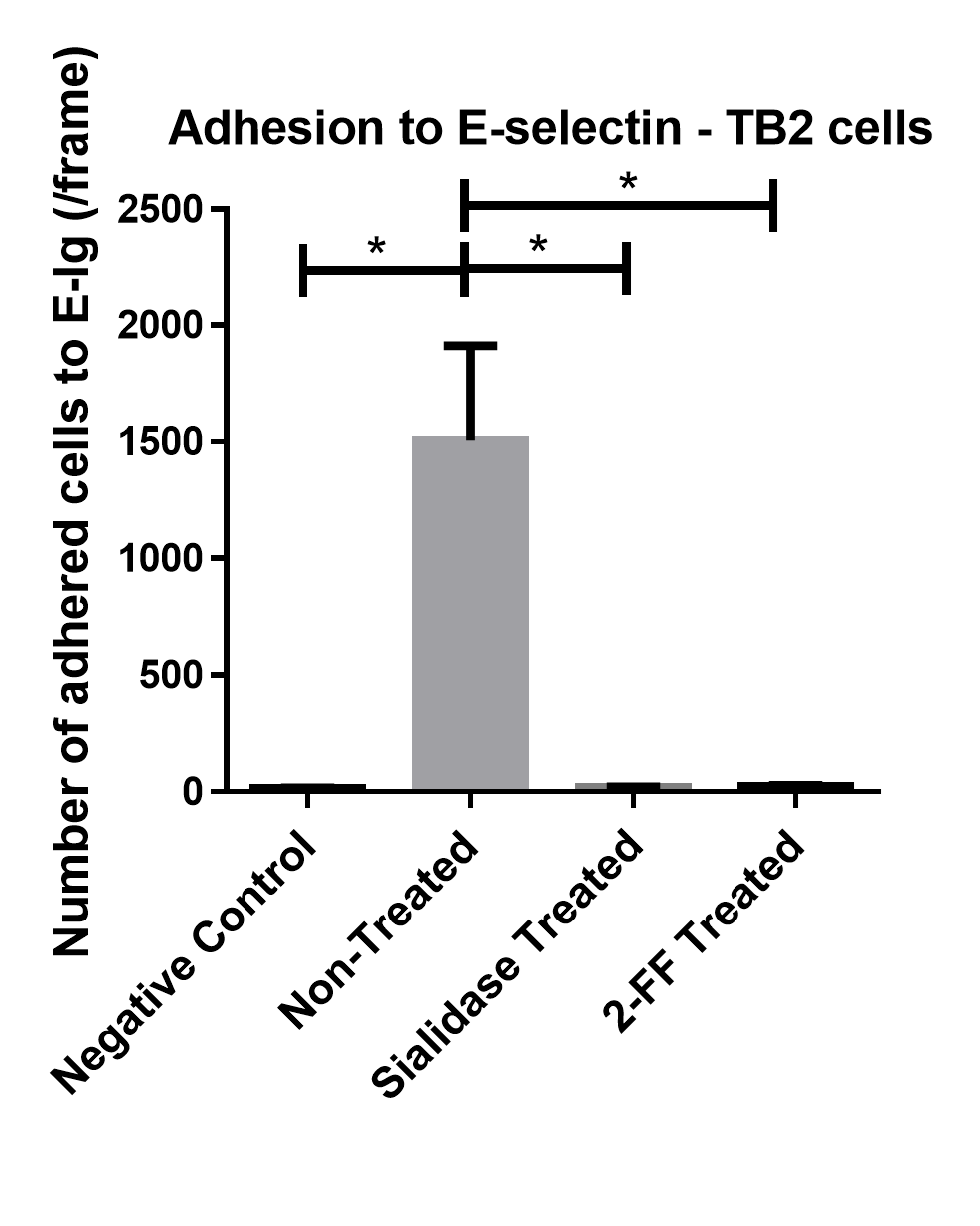

Supplement: Supplementary file 5 — Fig S5. TB2 primary breast IDC cells treated with 2‐FF compound loses functional E‐selectin ligands. [file MOL2-12-579-s005.tif]
