# Supplementary material for: Inhibition of fucosylation in human invasive ductal carcinoma reduces E‐selectin ligand expression, cell proliferation, and ERK1/2 and p38 MAPK activation
Source: Mol Oncol. 2018 Mar 30;12(5):579–93. doi: 10.1002/1878-0261.12163 (PMC5928367; doi:10.1002/1878-0261.12163)
Supplement: Supplementary file 6 [file MOL2-12-579-s006.pdf]

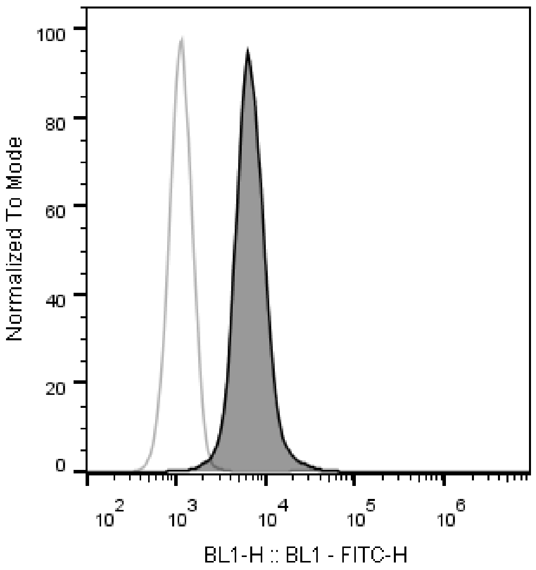

Fig S1: All CF1 primary cells express cytokeratins confirming their epithelial origin. CF1 primary cells were stained with anti-cytokeratin plus fluorescent secondary antibody after cell fixation and permeabilization (black filled pick) and analyzed by flow cytometry. Cells stained with just fluorescent secondary antibody were used as negative control (grey line).

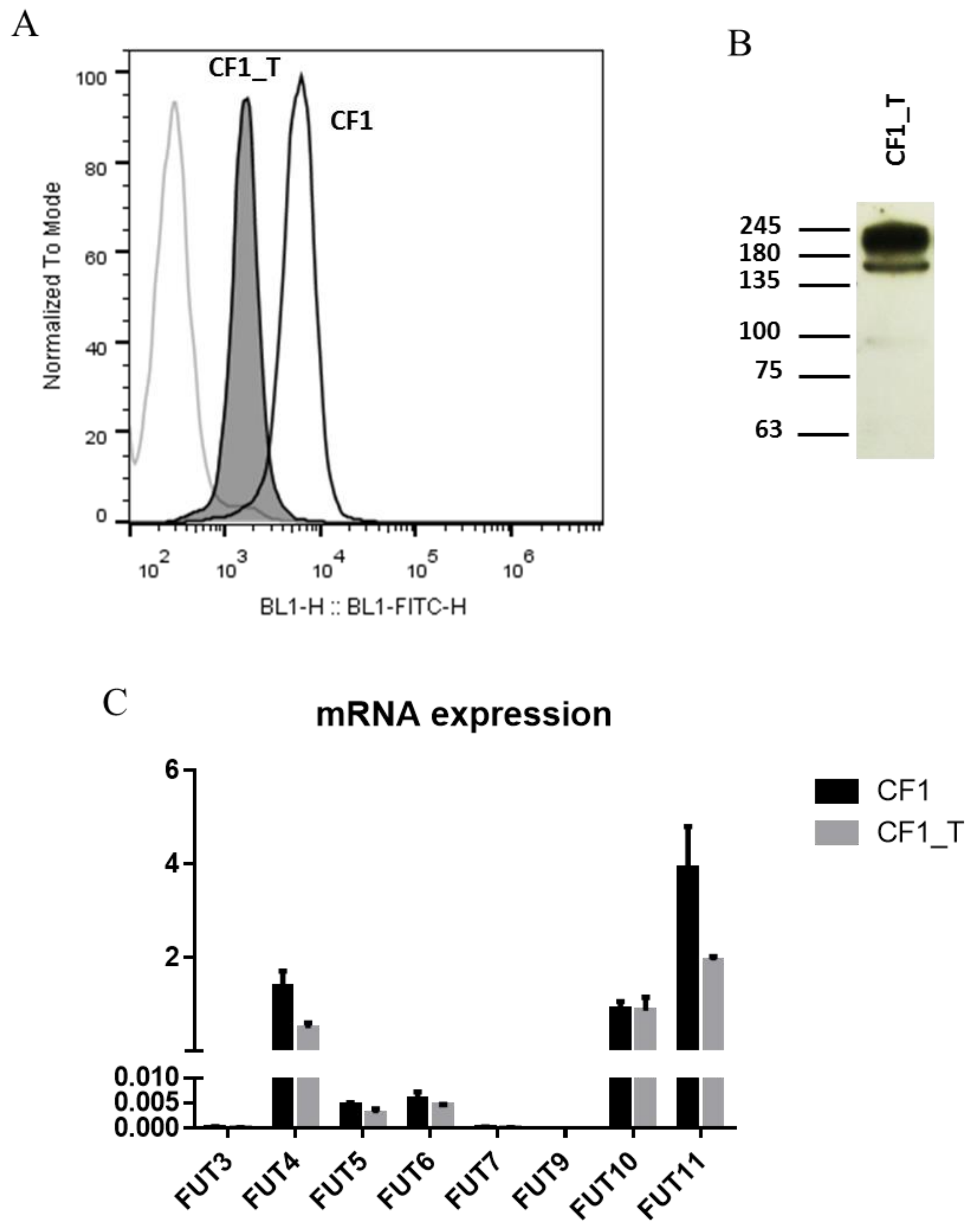

Fig S2: CF1_T cells continue to express E-selectin ligands and α1,3/4-fucosyltransferases after the immortalization process. A: Both CF1 primary cells (black line) and CF1_T cell line (filled pick) were stained with E-selectin chimera plus fluorescent secondary antibody in PBS with calcium and analyzed by flow cytometry. Cells stained in PBS with EDTA or with just fluorescent secondary antibody were used as negative control (grey line). **B:** CF1_T cell lysate was run by SDS-PAGE, blotted in PVDF membrane and stained using the monoclonal antibody HECA-452, directed to the lymphocyte-associated antigen (CLA), a cell surface glycoprotein that binds specifically to E-selectin. **C:** Gene expression of α1,3/4-FUTs in CF1primary breast cancer cells and CF1_T cell line analyzed by RT-PCR. Values correspond to the amount of RNA copies of each FUT per each 1000 copies of housekeeping genes (β-actin and GAPDH).

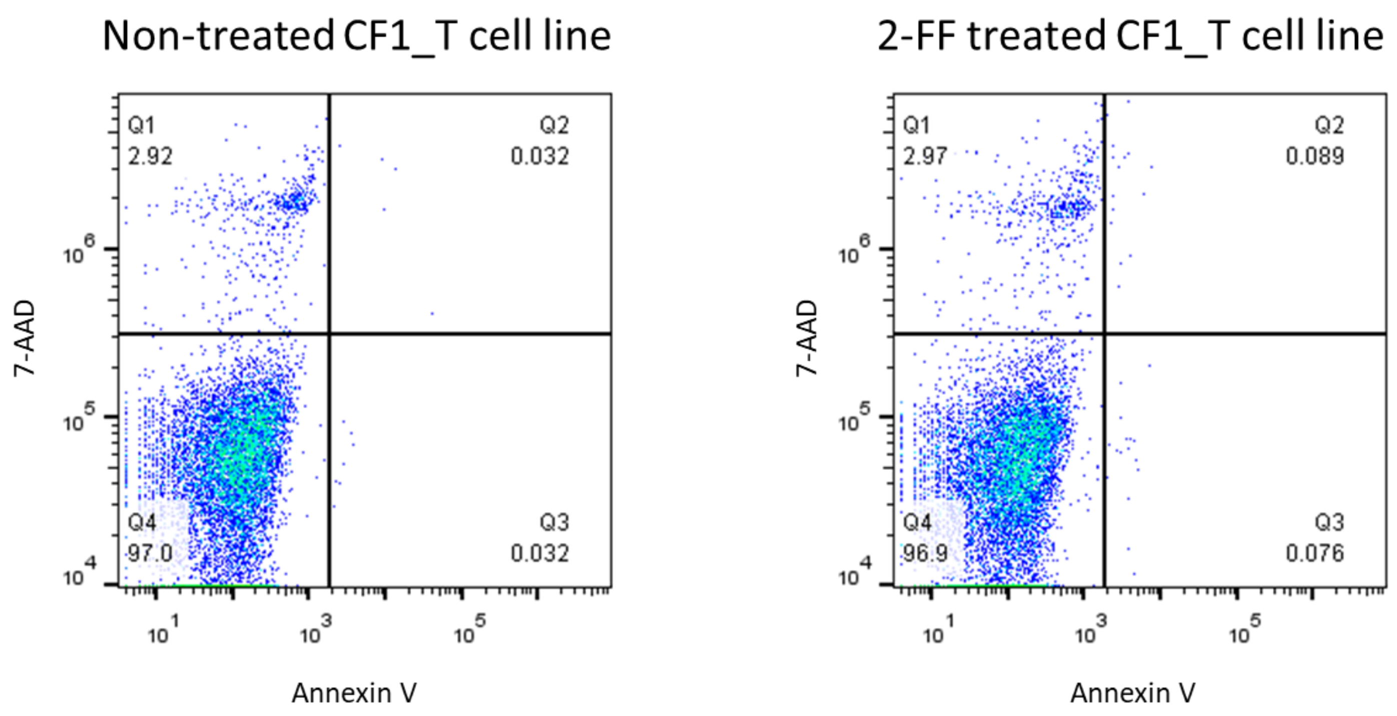

Fig S3: Treatment with 2-FF does not induce cell death of CF1_T cell line. Effect of 2-FF treatment on CF1_T cell viability was analyzed by flow cytometry. The CF1_T cells were treated or not with 2-FF for 18 days and their viability was evaluated using Annexin V (apoptotic cells) and 7-AAD (necrotic cells) staining.

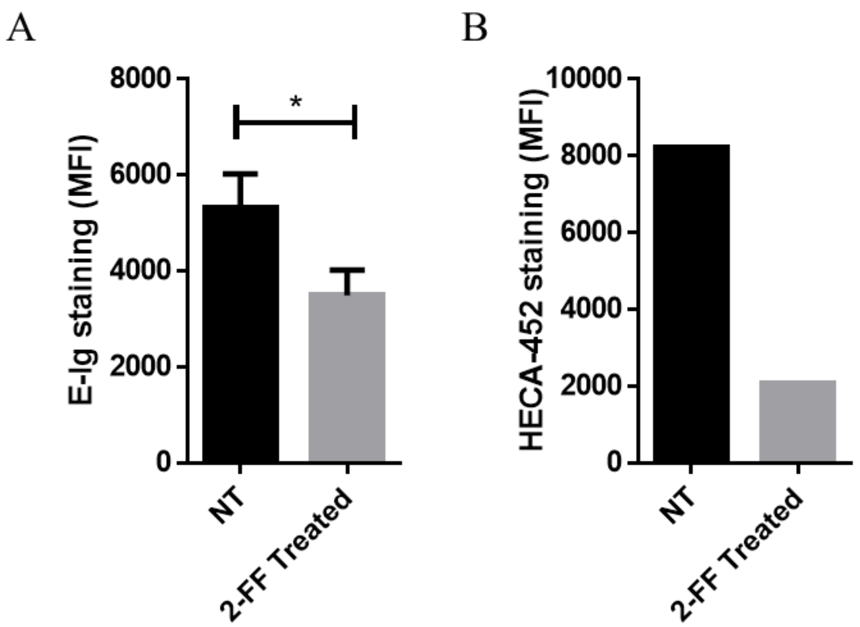

Fig S4: CF1_T cell line treated with 2-FF compound loses the expression of E-selectin ligands and sLe$^{X/A}$ glycans. The CF1_T cell line was treated with 1mM of 2-FF inhibitor (2-FF Treated) or not (NT), for 5 days, and then the expression of E-selectin ligands was analyzed using E-Ig chimera (**A**) and sLeX/A expression using HECA-452 mAb (**B**) by flow cytometry. The graphs show the mean fluorescence intensity (MFI).

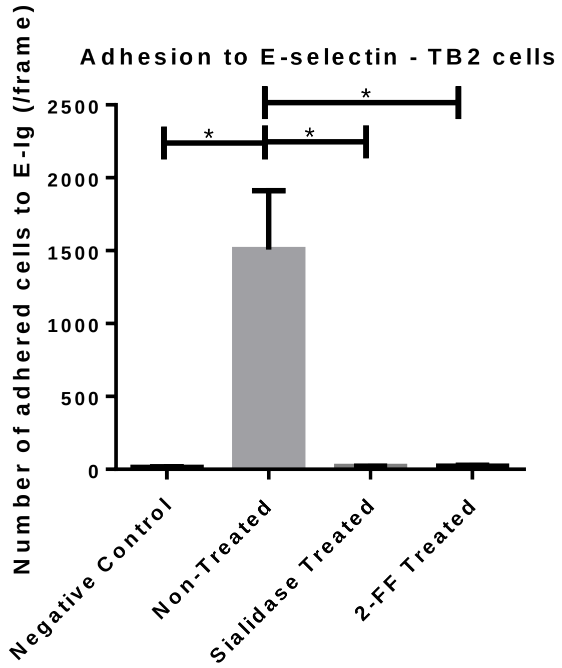

Fig S5: TB2 primary breast IDC cells treated with 2-FF compound loses functional E-selectin ligands. Effect of 2-FF and sialidase on the capacity of TB2 cells to adhere to E-selectin under flow conditions. The TB2 cells were treated or not with 2-FF for 5 days, or sialidase for 1h, and their capacity to adhere to E-Ig chimera was analyzed under flow conditions by an alternative Stamper-Woodruff assay. Cells were added in calcium buffer over an E-Ig spot and incubated with an orbital rotation at 80 rpm for 30 min, at 4ºC. Assays performed in EDTA buffer were used as negative control. (n=3; $p < 0.05$ (*))
